# Adapting Caring Contacts for Veterans in a Department of Veterans Affairs Emergency Department: Results From a Type 2 Hybrid Effectiveness-Implementation Pilot Study

**DOI:** 10.3389/fpsyt.2021.746805

**Published:** 2021-10-13

**Authors:** Sara J. Landes, Susan M. Jegley, JoAnn E. Kirchner, John P. Areno, Jeffery A. Pitcock, Traci H. Abraham, Sacha A. McBain, R. Sonia Singh, Mary J. Bollinger, Jacob Painter, Jack A. Woods, Nyssa D. Curtis, Donald E. Jones, Bridget B. Matarazzo, Mark A. Reger, Katherine Anne Comtois

**Affiliations:** ^1^Behavioral Health QUERI, Central Arkansas Veterans Healthcare System, North Little Rock, AR, United States; ^2^South Central Mental Illness Research Education and Clinical Center, Central Arkansas Veterans Healthcare System, North Little Rock, AR, United States; ^3^Department of Psychiatry, University of Arkansas for Medical Sciences, Little Rock, AR, United States; ^4^South Central VA Health Care Network, Ridgeland, MS, United States; ^5^Center for Mental Healthcare & Outcomes Research, Central Arkansas Veterans Healthcare System, North Little Rock, AR, United States; ^6^Rocky Mountain Mental Illness Research Education and Clinical Center, Rocky Mountain Regional Veterans Affairs Medical Center, Aurora, CO, United States; ^7^Department of Psychiatry, University of Colorado Anschutz School of Medicine, Aurora, CO, United States; ^8^VA Puget Sound Health Care System, Tacoma, WA, United States; ^9^Department of Psychiatry and Behavioral Sciences, University of Washington School of Medicine, Seattle, WA, United States

**Keywords:** veteran, suicide prevention, emergency deparment, caring contacts, implementation

## Abstract

Transitions in care, such as discharge from an emergency department (ED), are periods of increased risk for suicide and effective interventions that target these periods are needed. Caring Contacts is an evidence-based suicide prevention intervention that targets transitions, yet it has not been widely implemented. This pilot study adapted Caring Contacts for a Department of Veterans Affairs (VA) ED setting and population, created an implementation toolkit, and piloted implementation and evaluation of effectiveness. To inform adaptation, qualitative interviews were conducted with stakeholders. Data were used by an advisory board comprised of stakeholders, experts, and veterans to make adaptations and develop an implementation planning guide to delineate steps needed to implement. Key decisions about how to adapt Caring Contacts included recipients, author, content, and the schedule for sending. Pilot implementation occurred at one VA ED. Caring Contacts involved sending patients at risk of suicide brief, non-demanding expressions of care. Program evaluation of the pilot used a type 2 hybrid effectiveness-implementation design to both pilot an implementation strategy and evaluate effectiveness of Caring Contacts. Evaluation included qualitative interviews with veteran patients during implementation. VA electronic health records were used to evaluate VA service utilization in the 6-month periods immediately before and after veterans were delivered their first Caring Contact. Hundred and seventy-five veterans were mailed Caring Contacts and the facility continued adoption after the pilot. Participants were positive about the intervention and reported feeling cared about and connected to VA as a result of receiving Caring Contacts. This project developed an implementation planning process that successfully implemented Caring Contacts at one site. This can be used to further implement Caring Contacts at additional VA or community EDs.

## Introduction

Suicide is a growing public health problem, especially for military veterans. Although suicide rates have increased for veterans and non-veterans, rates for veterans are 1.5 times higher after adjusting for age and gender (1, 2). Suicide prevention remains a top priority for the Department of Veteran Affairs (VA) (3). Transitions in care are critical periods of suicide risk (4). Most deaths by suicide take place within 30 days of discharge from the hospital or emergency department (ED), with most occurring within a week (5–7). In a large meta-analysis of 100 studies, the suicide rate 3 months post discharge was ~100 times the global suicide rate (8). Given this, national suicide prevention initiatives have focused on transitions in care to improve suicide prevention (9–13).

Caring Contacts (CC) is an evidence-based suicide prevention intervention that involves sending patients at risk of suicide brief, non-demanding expressions of care over a period of time (usually 1 year) (14–18). The most frequently used CC mailing schedule is 1, 2, 3, 4, 6, 8, 10, and 12 months following discharge from inpatient or outpatient mental health and/or refusal of services (19). CC have been sent via postal mail, email, and text message.

In randomized controlled trials (RCTs), CC resulted in significant decreases in rates of suicide mortality (14, 15). Suicide attempts and ideation were significantly reduced at 1- and 2- year follow-up (16–18). CC is feasible and acceptable in veteran and military samples. Veterans on a psychiatric inpatient unit reported CC would be helpful, and a majority identified postal mail as their preferred modality (20). In an RCT of CC via text message with service members, Comtois et al. (21) found that as an adjunct to outpatient mental health, CC resulted in reduced odds of suicidal ideation and suicide attempt. In an RCT of CC via email with service members and veterans, there were no adverse events and no difference between those who received CC and the control group on death, suicide attempts, or psychiatric readmission (22).

Although clinical practice guidelines recommend CC (23), the intervention has not been implemented broadly, nor has its implementation been evaluated in practice. This lag in implementation is not uncommon. As early as 2001, the Institute of Medicine identified a “quality chasm” that exists between the development of new clinical innovations and practices and their implementation into routine clinical care (24). Researchers have estimated that this chasm or gap is ~17 years, with only half of evidence-based practices being implemented into clinical care at all (25). In addition, effort is needed to support the systemic uptake of evidence-based practices in healthcare settings. Further, as evidence-based practices move from efficacy to effectiveness to sustained application, quality gaps and lost fidelity to the practice may occur (26). Implementation science has emerged as a field to rigorously study how to address the quality chasm and improve the implementation of evidence-based practices into routine clinical care. Implementation science has been defined as the scientific study of methods and strategies to shift evidence-based practice and research into regular, routine use (27). Implementation science methods and strategies can be used create a plan for successful implementation, identify implementation strategies to support implementation, create and adapt tools specific to the needs of the setting and population, and evaluate implementation efforts (26).

The current project was conducted to determine how to implement CC for veteran patients in a VA ED using implementation science methods. The aims were to (1) adapt CC for use in VA ED settings, (2) conduct an implementation pilot, and (3) create an implementation toolkit for facilitating the spread of CC.

## Materials and Methods

### Overview

The pilot protocol was previously published (28). In summary, this project included a planning phase and an implementation pilot in a VA ED. The planning phase included determining data sources, creating an advisory board, and creating an implementation toolkit. We conducted qualitative interviews with ED staff and other key stakeholders on what was needed to adapt and implement CC for a VA ED setting and to identify barriers to and facilitators of implementation. The advisory board adapted CC and planned implementation, informed by an implementation planning guide.

We used the integrated Promoting Action Research on Implementation in Health Services (i-PARIHS) (29) framework to guide implementation and evaluation. The i-PARIHS framework proposes that successful implementation of a practice is the result of facilitation with recipients in the context of the inner and outer setting. Facilitation is the implementation strategy, with designated individuals serving as facilitators who activate implementation by assessing and responding to characteristics of the recipients of the innovation within their settings.

### Setting

The setting was an ED at a large VA medical center in a Southern, rural state. This ED employs 15 physicians, two midlevel providers, 56 nurses, and four social workers. An average of 2,000 patients are seen monthly; ~200 for mental health reasons.

### Planning Phase Methods

#### Participants

A champion for CC implementation in the ED was identified by facility leadership based on interest and role. The champion, the ED Nurse Manager, helped move the project forward, including engaging and training stakeholders. The champion recruited key clinical staff for qualitative interviews.

#### Advisory Board

We established an advisory board consisting of stakeholders from the ED, facility, and regional network, as well as experts in implementation and CC and veteran representation. See Table 1 for full list. The advisory board served two functions: (1) adapting CC for implementation in a VA ED and (2) implementation planning for the pilot. The advisory board met five times over 6 months.

**Table 1:** Advisory board members (in alphabetical order).

•	Ambulatory Care Unit Manager
•	Associate Director of Patient Care Services
•	Associate Nurse Executive for Research
•	Caring Contacts Expert
•	ED Health Tech
•	ED Nurse
•	ED Nurse Manager
•	ED Social Worker
•	Health System Specialist for Innovations
•	Implementation Expert
•	National Lead for Suicide Risk Identification Strategy Implementation
•	Suicide Prevention Coordinators (2)
•	Veteran
•	Regional Mental Health Lead

#### Implementation Planning Guide

The research team and advisory board adapted the Behavioral Health QUERI implementation planning guide (30) for CC in the ED. The implementation planning guide is designed to be a living document that includes actionable items; current status, potential barriers, and notes; a plan with a timeframe; who is in charge; and metrics along with defining success for each key task. See Supplementary Materials.

#### Measures

Qualitative interviews with staff were conducted by one of two members of the research team with qualitative interviewing experience and expertise in implementation (JEK) and CC (SJL) using semi-structured interview guides informed by i-PARIHS. All interviews were audio recorded and transcribed verbatim. Interview guides provided data about CC and its effectiveness. Participants were asked if CC would be useful for veterans in the ED and how veterans should be identified to receive CC. Participants were asked their opinions on content, schedule, logistics, and resources needed to send CC, as well as potential barriers and facilitators of implementation. Interviews averaged 20 min in length.

#### Data Analysis

Rapid analytic techniques (31) were used to quickly produce findings for use by the study team and advisory board. A qualitative analyst reviewed transcripts and summarized content using a template with domains based on implementation goals. Categories were developed within domains as content was summarized. Templates were aggregated in a summary template. To establish rigor, individual templates were audited by a second analyst for accuracy and completeness. A rubric was developed to define template domains and ensure consistency in how template content was organized, akin to establishing agreement among coders.

#### Staff Perspectives

We conducted qualitative interviews with 11 stakeholders: five ED nurses, one ED social worker, one ED physician, one ED health tech, two Suicide Prevention Coordinators, and one outpatient mental health provider. Overall, they had positive perceptions of CC. One said, “I'm a veteran. I feel like that would be a wonderful thing to let people to know that it's not just from a large organization, but there are people here who actually care about you.” See Table 2 for a summary of stakeholder feedback.

**Table 2:** Feedback from stakeholder interviews about logistics of implementing Caring Contacts (CC) in an ED.

**Category of Feedback**	**Feedback**
Who is appropriate to receive CC	Send to everyone with “self-harm or suicidal ideations” Concern about patients who frequently visit ED and logistics of sending to them
How to identify patients for CC	Use screening tools (e.g., PHQ-9 item 9, Columbia-Suicide Severity Rating Scale) Have providers identify who is appropriate (concern about burden) Use the mental health report from the ED Integrative System
Schedule for sending	Consensus to send first CC sooner than 1 month after discharge Suggestion to send CC on Veterans Day
Content of Caring Contacts	Include Veterans Crisis Line information as well as a local number Wording suggestion: “It was an honor to serve you.”
Author of Caring Contacts	Suggestions: • ED Nurse or Social worker • Suicide Prevention Coordinator • ED provider who conducted screen for suicide risk • Combination of ED provider & Suicide Prevention Coordinator Do not use ED physician or psychiatry residents (they often change)
Logistics - Concerns	Concern about veterans who are homeless and without a mailing address Concern about up-to-date mailing addresses
Logistics – Who will mail, track, and document	Suggestions: • Social worker • Suicide Prevention Coordinator •Delegate to non-emergency staff or non-provider Recommendation to centralize the process (e.g., run weekly report, send all at once)

#### Key Decisions Made

Guided by the implementation planning guide, the advisory board made decisions about adaptation of CC and the implementation plan informed by their expertise, stakeholder interview data, veteran input [from advisory board members and previous data (20)], and expert opinion from implementation and CC experts. A central goal of this project was to determine how to implement CC in a way that would minimize provider burden and allow scale-up and spread. Therefore, the advisory board also sought to align and build upon existing VA initiatives and resources, such as the national VA suicide risk identification strategy (32) and dashboards supporting suicide prevention. Here we describe decisions made about adaptations and the implementation plan and factors that impacted decisions.

Who receives CC and how to identify? The advisory board selected the VA-required screen for suicide risk in ED triage as the method for identifying veterans to receive CC (33). Item 9 from the Patient Health Questionnaire-9 (PHQ-9) (34) was initially used as the primary screener; VA later changed this to the Columbia-Suicide Severity Rate Scale Screener (C-SSRS) (35). To have a broad reach, endorsement of at least one of the eight items on the C-SSRS was considered a positive screen for CC. The screen is part of the national ED triage note, which generates data for an existing dashboard which was used to identify eligible veterans and export a patient mailing list to facilitate outreach. If a veteran screened positive for suicide risk across multiple ED visits, CC were sent based on the first date and the schedule was not restarted or changed.

Schedule for sending. The advisory board chose the following mailing schedule for 11 cards: 1 week after discharge from the ED regardless of disposition status (e.g., to inpatient psychiatry, to home); months 1, 2, 3, 4, 6, 8, 10, and 12; veteran's birthday month; and Veterans Day. This schedule is in alignment with those most frequently used in research, with the addition of a card within 1 week of discharge given the higher risk during that time period. Veterans' birthday month and Veterans Day were added based on input from veterans and other stakeholders. There was discussion about sending CC after discharge to home, but the level of tracking required to do this was not feasible given the tracking system available.

Content and author. The advisory board chose to have unique messages for each card in the schedule. Research staff with CC expertise generated content for each card and a variety of layout options and logos. Content and layout were revised based on feedback from the advisory board, which included veterans. Templates were created for ease and consistency. The advisory board determined that the signatory of all cards would be the ED nurse manager (also the local CC champion) and the ED team, written as “Jenny Smith^1^, RN and Your Emergency Department Team.” This reduced the need to pull additional data from the electronic health record (EHR; i.e., nurse who completed screen) and streamlined the point of contact.

Logistics. The group finalized logistics of sending CC and created a standard operating procedure. This included running a weekly dashboard report that included ED suicide screening data; the report included name, gender, address, and date of birth. This standard operating procedure also included steps to check for patients who were already receiving CC so as not to restart or duplicate the intervention. Research staff tested various methods for preparing CC and selected mass-producing cards through the facility print shop. In consultation with facility leadership, the advisory board determined that a facility program support assistant (staff member with clerical duties) would administer CC and carry out the tasks of generating the weekly report, mailing, and documenting each card in the EHR. Initially a research team member completed these tasks and then facilitated transition to the designated staff member. Procedures were developed to organize responses to patient replies to CC (e.g., expression of thanks, indications of risk, requests to cease sending CC).

Training. The advisory board developed a training plan for the facility and a training plan template for use at other sites. Training was organized into four phases: (1) staff directly impacted by CC (e.g., ED staff, nursing, phone operators who might receive calls related to CC); (2) staff likely to interact with veterans receiving CC (e.g., primary care, mental health); (3) broader employee population; and (4) outside stakeholders (e.g., veteran service organization representatives). The team developed brief handouts, FAQs, and PowerPoint presentations to support training and education.

#### Implementation Toolkit

Informed by the planning process, the research team developed a CC implementation toolkit to facilitate spread. This toolkit is located on a VA intranet site to allow access to any VA employee who wishes to implement CC. The toolkit was built using the Agency for Healthcare Research and Quality (AHRQ) Publishing and Communications Guidelines (36) and evidence-based toolkit development recommendations (37). It includes the implementation planning guide, leadership briefings, educational materials (e.g., brochures, PowerPoint presentations with notes, FAQs, research summaries), CC templates, and standard operating procedures.

### Implementation Pilot Methods

The implementation pilot occurred in one VA ED and CC were provided as a component of usual care. Facilitation was used to support implementation. Program evaluation used a type 2 hybrid effectiveness-implementation approach (38, 39) to both evaluate piloting of an implementation strategy and gather data on CC's effectiveness. Evaluation was guided by RE-AIM (40) to examine reach into the target population, effectiveness of CC, patient experience of receiving CC, adoption by the setting, and implementation fidelity.

#### Participants

All veterans seen in the ED who screened positive for suicide risk received CC.

#### Interview Recruitment

Veterans who were mailed CC for at least 6 months were recruited for qualitative interviews. This timeframe allowed for veterans to have been mailed at least six CC (week 1, months 1, 2, 3, 4, 6) and possibly two more (Veterans Day, birthday month), which allowed for them to likely have received enough cards to have an opinion about CC.

Recruitment letters invited veterans to participate in a project to help VA better understand follow-up contact after the ED and offered $50 reimbursement for their time. Based on a goal of five qualitative interviews and previous response to recruitment letters at this facility, we mailed 83 letters. However, 18 veterans called about participation and we were able to accommodate additional interviews. Ten veterans were interviewed.

#### Implementation Strategy

Facilitation was selected as the implementation strategy to support implementation of CC given its flexibility and evidence base. Facilitation is a “process of interactive problem solving and support that occurs in the context of a recognized need for improvement and a supportive interpersonal relationship.” Facilitation has been used in VA to implement several clinical interventions (41, 42). Facilitation was provided by the PI (SJL) and project coordinator (SMJ). See Table 3 for a list of facilitation strategies and activities that were used.

**Table 3:** Facilitation strategies and activities as recorded in meeting notes.

**Strategy**	**Activities**
Facilitate local change agent participation	• Helped CC champion engage their facility and impacted providers • Assisted CC champion in creating an implementation planning group • Guided group in use of implementation planning guide • Identified individual(s) responsible for logistics of CC
Conduct provider education	• Provided briefings to medical center leadership to ensure they are aware of and supportive of CC • Educated CC staff and providers on CC program components • Assisted CC champion in developing a site training plan
Facilitate stakeholder engagement	• Were available for consultation about the program to regional and local leadership as needed and as identified by local change agents
Program adaptation	• Guided implementation planning group in making adaptations to CC program
Problem resolution	• Assisted with logistic issues (e.g., mailing, obtaining a phone line)
Facilitate performance monitoring and feedback	• Created reports of CC staff and provider activity • Presented reports to CC staff and local leadership
Conduct formative evaluation	• Helped site identify possible barriers and facilitators to implementation and address them
Facilitate program marketing	• Supported marketing activities
Link with experts and/or resources	• Connected to experts in CC, suicide risk screening, database function

#### Intervention: Caring Contacts

**Figure 1:**
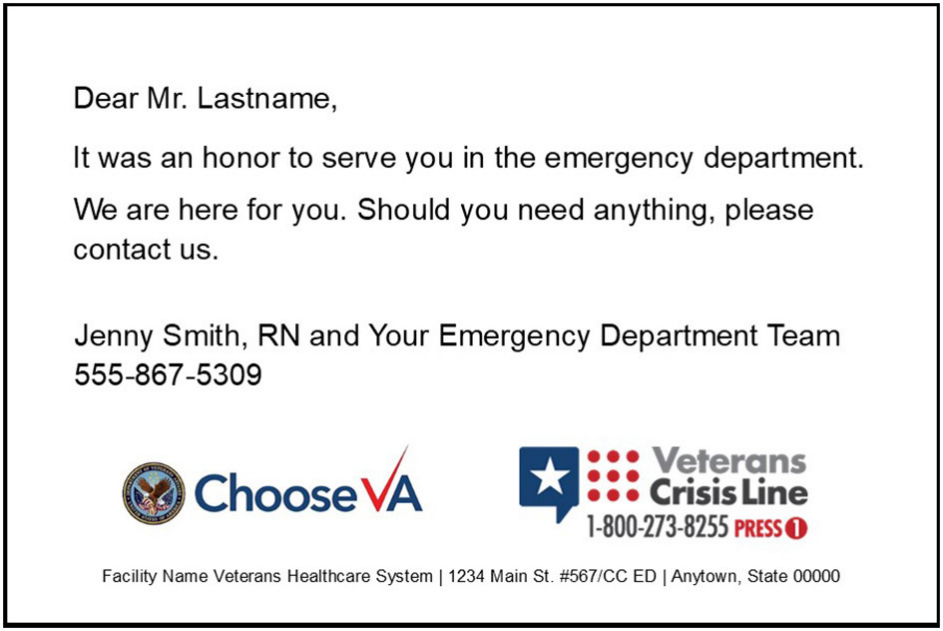
Sample Caring Contact sent 1 week after discharge from ED.

As described above, the advisory board adapted CC for the VA ED setting. Messages were sent on behalf of the ED Nurse Manager and the ED Team and documented in the EHR. CC were printed on a flat card and sent in a light blue sealed envelope. See Figure 1 for a sample card and Table 4 for the main content of each card.

**Table 4:** Content of each CC based on schedule for sending.

**Schedule**	**Message**
Week 1	It was an honor to serve you in the emergency department. We are here for you. Should you need anything, please contact us.
Month 1	We value your health and are thinking of you. We are here for you. If you wish to contact us, we would be happy to hear from you.
Month 2	We care about you and hope this message finds you well. Should you need anything, please contact us.
Month 3	“*Who kept the faith and fought the fight, the glory theirs and the duty ours.” Wallace Bruce* We value your health and are honored to serve you in the emergency department.
Month 4	Sending positive thoughts your way today. However, things are going, we are here for you.
Month 6	We hope things are going well for you. If you wish to contact us, we would be happy to hear from you.
Month 8	We are thinking of you. We are here if you need us. Should you need anything, please contact us.
Month 10	We hope you are doing well. We are honored to serve you at the VA and are here if you need us.
Month 12	We continue to wish you health and happiness. This will be our last card to you. Although you will no longer receive cards from us, we are still here for you.
Veterans Day	To you who has served our country, we honor you today and every day. Thank you for your service. We are honored to serve you at the VA.
Birthday	All of us at Facility Name Emergency Department wish you a happy birthday and good health in the years to come!

A dedicated phone line was established and included on the CC. Consistent with VA policy, the phone line had a message indicating when someone is available to return their call and to call 911 or the Veterans Crisis Line if they are at immediate risk and that if they called regarding a card from the ED, they have called the correct number. The ED Nurse Manager checked the voicemail daily and a dedicated postal mailbox and responded to messages in a timely manner consistent with VA policy.

#### Measures

We used RE-AIM (40) as our analytic framework to examine five dimensions: reach into the target population, effectiveness of the intervention, adoption by the setting, implementation consistency or fidelity, and maintenance over time. Table 5 summarizes how we defined each dimension. See protocol paper for more detail (28).

**Table 5:** Dimensions measured with their definition and method of obtaining data.

**Measure**	**Definition**	**Data source**
Reach	Number and % of eligible veterans receiving CC	Administrative data
Adoption	Continued use of CC after implementation	Administrative data
Implementation fidelity	Date sent and adherence to schedule Content of CC and whether appropriate template was used (i.e., the right card for each time point)	Chart review
Cost	Cost of implementing CC Cost of providing CC	Administrative data
Staff perspective	Key informant interviews focused on staff perspective of CC	Qualitative interview
Effectiveness: suicide related behavior	Self-directed violence rate	Administrative data
Effectiveness: service utilization	Outpatient mental health encounters Outpatient health/other encounters Inpatient admissions Emergency department visits	Administrative data
Veteran perspective	Key informant interviews focused on veteran perspective of CC	Qualitative interview

*As this was a pilot with a brief timeline, we did not measure maintenance*.

A facilitation time tracking log (43) was adapted for the project to collect facilitator time devoted to implementation activities. Given the significant burden time tracking would place on the CC champion and specialist, percent effort devoted to the project was used to account for their time.

Qualitative interviews with veterans were conducted by telephone by two members of the research team with experience with qualitative interviewing (THA, NDC) using semi-structured interview guides informed by i-PARIHS. All interviews were audio recorded and transcribed verbatim. Interview guides included 13 survey questions using a Likert-type scale (1 = completely disagree, 5 = completely agree); see **Table 7** for items. Open ended questions included how many cards were received, if recipient had replied to any cards, if any card was more meaningful, if cards arrived at an important time, if getting a card bothered them, thoughts about receiving the message by text message, and suggestions for improvement. Interviews averaged 54 min in length.

#### Data Analysis

Quantitative. We used VA EHR data to evaluate healthcare utilization in the 6 months prior to receiving CC and in the 6 months immediately following the sending of the first CC from the ED. We tested the feasibility of collecting data and testing for pre-post changes using paired *t*-test analyses. Unless noted otherwise, the time period of reference was May 17, 2019 to May 17, 2020.

Qualitative. As in the planning phase, we used rapid analytic techniques (31).

## Implementation Pilot Results

### Reach

During the 1-year timeframe, there were 709 positive suicide screens. Some patients presented to the ED more than once and were thus screened multiple times; there were a total of 532 unique patients. CC were not sent to 49 patients due to no mailing address/homelessness and eight for clinical reasons (e.g., paranoia). Of the 532 unique veterans with positive screens, 475 (89%) were mailed CC.

### Adoption

As of 6-months post implementation, the staff person continued to send CC.

### Implementation Fidelity

We sampled the 90-days post implementation to evaluate implementation fidelity. We tracked whether each CC was sent according to schedule (defined as within 7 days of target date; Y/N) and whether the appropriate template was used (Y/N). Of the 169 CC sent in this timeframe, 110 (65%) were sent on schedule and 59 (35%) were not. Those not sent on schedule were sent 1–11 days past the timeframe. When focusing on the first card sent for week 1, the highest risk period, 89 of the CC sent during the sampled timeframe were week 1 cards. Of the week 1 cards, 77 (87%) were sent on schedule and 12 were not (13%). All (100%) used the appropriate template.

### Cost

It was feasible to collect cost data related to implementation and provision of the intervention. Facilitators spent ~5 h per week over 8 weeks supporting implementation. The CC champion (ED Nurse Manager with supervisory authority over ED nursing) estimated that their dedicated time was 10% over 6 months for planning and implementation. Once implementation was complete, they had 0% dedicated time; their main task was to check the voicemail and respond to calls. The CC specialist (program support assistant) had 50% protected time to coordinate the CC program. In addition to personnel time, material costs were collected; this included the cost of printing CC cards using the facility print shop, envelopes, and address labels. Material costs were $2.45 per veteran for all 11 cards. Postage costs were $6.05 per veteran for all 11 cards using the 2020 postal rates. Total material and postage costs to send all 11 cards over the course of 1 year was $8.50 per veteran.

### Effectiveness

The pilot effectiveness cohort consisted of 348 veterans who screened positive for suicide risk and were mailed at least six CC between May 17, 2019 and May 17, 2020. The sample was 90.2% male (314/348) and the mean age was 52.5 years (*SD* = 14.5).

### Effectiveness: Suicide-Related Behavior

It was feasible to identify this data. However, there were too few instances of suicide-related behavior reported during this time frame for analysis purposes.

### Effectiveness: Service Utilization

It was feasible to identify this data. Paired *t*-tests of utilization measures indicated that veterans receiving CC were seen in the ED significantly less often in the 6-month post period as compared to the previous 6 months (2.5 to 1.3, *p* < 0.001), had fewer outpatient mental health visits (1.0 to 0.6, *p* < 0.05), and had fewer inpatient admissions (1.4 to 0.7, *p* < 0.001). Additionally, non-mental health outpatient visits decreased from 18.5 to 15.1 (*p* < 0.05). The mean number of no-show appointments did not differ between pre and post (2.3 to 2.6, *p* > 0.05). See Table 6.

**Table 6:** Change in service utilization.

**Measure**	**Pre (SD)**	**Post (SD)**	**Paired *t*-test, *p*-value**
Emergency department encounters per patient	2.5 (2.6)	1.3 (2.8)	*t*_(347)_ = 7.78, *p* < 0.001
Mental health encounters per patient	1.0 (1.5)	0.6 (2.5)	*t*_(347)_ = 2.97, *p* < 0.05
Non-mental health encounters per patient	18.5 (26.3)	15.1 (24.1)	*t*_(347)_ = 2.87, *p* < 0.05
Inpatient admissions per patient	1.4 (1.5)	0.7 (1.4)	*t*_(347)_ = 8.28, *p* < 0.001
No show appointments	2.3 (3.5)	2.6 (3.1)	*t*_(347)_ = −0.3, *p* > 0.05

### Response to CC

No veterans responded to CC by postal mail. Four calls were received on the dedicated phone line; all were expressions of thanks for the cards. No requests to cease sending CC were received.

### Veterans' Perspectives

Participants completing qualitative interviews were (*N* = 10); they included eight men and two women. They ranged in age from 44 to 67 (*M* = 53.9) years and identified as White (*n* = 7) and Black (*n* = 2). See Table 7 for summary of participant responses. Veteran responses to survey questions indicated that they felt that CC brightened their day (75%) and were helpful (75%). Two of the participants interviewed did not recall receiving the cards and did not answer the survey questions. They participated in the qualitative interview portion to assess their opinion about CC.

**Table 7:** Summary of veteran responses to survey questions about Caring Contacts mailed to them (*n* = 8).^*^

	**Completely disagree**	**Somewhat disagree**	**Neither agree nor disagree**	**Somewhat agree**	**Completely agree**
	***n* (%)**	***n* (%)**	***n* (%)**	***n* (%)**	***n* (%)**
I liked receiving letters from the ED nurse over the past few months.	0 (0%)	0 (0%)	1 (12.5%)	1 (12.5%)	6 (75%)
I felt the cards were intrusive.	8 (100%)	0 (0%)	0 (0%)	0 (0%)	0 (0%)
I wish I had received more cards.	3 (37.5%)	1 (12.5%)	3 (37.5%)	1 (12.5%)	0 (0%)
I felt that I received too many cards.	6 (75%)	0 (0%)	0 (0%)	1 (12.5%)	1 (12.5%)
The cards made a positive difference in my life.	1 (12.5%)	0 (0%)	1 (12.5%)	3 (37.5%)	3 (37.5%)
The cards gave me a sense of hope.	1 (12.5%)	2 (25%)	1 (12.5%)	2 (25%)	4 (50%)
The cards helped me to cope.	2 (25%)	0 (0%)	2 (25%)	1 (12.5%)	3 (37.5%)
I felt a sense of connection to the ED nurse because of the cards.	1 (12.5%)	0 (0%)	0 (0%)	1 (12.5%)	6 (75%)
I felt a sense of connection to the clinic staff because of the cards.	0 (0%)	1 (12.5%)	1 (12.5%)	1 (12.5%)	5 (62.5%)
I think the cards were helpful.	1 (12.5%)	0 (0%)	0 (0%)	1 (12.5%)	6 (75%)
The cards brightened my day.	1 (12.5%)	0 (0%)	0 (0%)	1 (12.5%)	6 (75%)
A card was seen by someone whom I did not want to know about my involvement in the project.	7 (87.5%)	0 (0%)	1 (12.5%)	0 (0%)	0 (0%)
I would recommend sending these cards to others who were in my situation.	0 (0%)	0 (0%)	0 (0%)	2 (25%)	6 (75%)

*Two of the ten participants interviewed did not recall receiving the cards and did not answer these questions*.

Participants were largely enthusiastic about CC during interviews. One male veteran stated, “I felt like my life matters to ‘em, you know?” Another male veteran described how receiving CC made him feel, stating, “I appreciate them sending the cards out though to check on me because, you know, I have several suicidal attempts. That made me feel good, that “Hey, I'm being thought about”.” One female veteran described, “It was … personable and seems like they really took the time out to care.” Most participants had better recollection of how the messages made them feel than specifics about message content or how many CC they received. This was illustrated by one male veteran, who when asked about the card messages stated, “It was mainly just wishing me well and a number to call if I wanted to.”

Few negative impacts or perceptions were described during the interviews. One male veteran felt that printed cards were too impersonal and “seemed … computer generated.” At least one veteran was confused by the purpose of the cards and thought they were “thank you” cards.

Numerous positive impacts or perceptions were identified. CC appeared to enhance some veterans' sense of connection to VA, to the ED nurse, and/or to ED staff. One female veteran said, “It made me feel really important. Like I belonged to the VA.” In some cases, the messages mitigated negative perceptions about the VA. One female veteran stated, “Sometimes you feel like you're a number there. And I didn't feel like a number. I felt like they truly cared … because they spent the time to follow-up and do that.” Another participant likewise stated, “I was surprised they were concerned.” These statements were supported by data from the survey questions indicating that CC made most participants (75%) experience a sense of heightened connection the VA.

Feedback from participants suggests that CC could fill an important gap for socially isolated veterans. For example, one male veteran said, “But if you're not in any program, if you just live out here in the real world… Around the holidays, I get kind of left out because I live alone, you know.” A female veteran said, “It was nice to get something … I'm an older person and a lot of people in my family have passed on and I've lost some close friends and stuff so…your support circle starts to shrink.”

## Discussion

This project sought to adapt CC for use in VA ED settings, conduct an implementation pilot, and create an implementation toolkit for facilitating the spread of CC. The planning phase allowed for collection of key stakeholder feedback about the intervention and its fit with the setting and veterans. Use of an advisory board with key stakeholders and CC experts allowed for adaptation of CC to the setting and recipient while keeping aligned with what is known about CC via research and capitalizing on existing resources and national initiatives. While this facility was not sending CC in other settings at the outset of the project, suicide prevention staff were familiar with the intervention and participated in planning. Inclusion of veterans and multiple stakeholders allowed for iterative changes to CC during planning. The advisory board used and adapted the Behavioral Health QUERI implementation planning guide to adapt the intervention and plan implementation. This resulted in a tool and process that will support spread of implementation to other sites. This implementation planning guide could also be used to adapt CC for other settings and populations, inside and outside VA.

The implementation pilot was successful in implementing CC in a VA ED, and the process resulted in creation of additional tools that were added to the implementation toolkit. Research staff were able to handoff to facility staff; adoption was maintained over the 6-month observation period. Of note, as of writing of this manuscript, adoption had continued for 20 months and the program continued to be in place. It has been maintained during the COVID-19 pandemic. CC were sent to a high percentage of eligible veterans (89%), indicating that the intervention had significant reach. Implementation fidelity was good, with 65% of cards sent within 7 days of the target date and 100% sent with the correct template. When focusing only on the first card, fidelity to the schedule was higher (89%). The lower fidelity of schedule was likely impacted by the ebb and flow of work duties. This may have less importance for monthly cards, as opposed to the first card sent 1 week after discharge, when suicide risk is highest.

For pilot analyses, we used a sample of veterans who had been mailed at least 6 months of CC. We compared the 6-months prior to receiving the first CC to the 6-months following. Therefore, we have an indication of the impact of the initiation of CC and did not evaluate the impact of the full intervention (i.e., all 11 cards). There are several other limitations to this analysis. Changes in service utilization may be related to being mailed CC, the passing of time, or other intervention. Individuals identified as high-risk in the ED may be more likely to have received a variety of services in the prior 6-months. This cohort includes those who received CC in 2020, so the COVID-19 pandemic may also have impacted utilization as some may have been less likely to present for services despite the availability of services.

VA's Suicide Prevention Applications Network (SPAN) data was originally identified as the primary source for obtaining suicidal behavior data. Since the time of the pilot, VA has transitioned to reporting suicidal behavior via the Suicide Behavior and Overdose Report (SBOR) and the Comprehensive Suicide Risk Evaluation (CSRE). Future efforts will utilize the SBOR and CSRE as the primary suicide behavior data sources.

CC were received positively by the veterans interviewed. Participants in the interviews described feeling that they mattered and feeling more connected to VA as a result of receiving CC. Participants had difficulty recalling the content or number of cards and had better recollection of how receiving the cards made them feel. Veterans highlighted that this intervention could be particularly helpful for those who are socially isolated.

Staff perspectives post-implementation were not collected due to limited staff availability during the COVID-19 pandemic. However, we have anecdotal evidence that the nurse manager was pleased with the project and CC's positive effect on veterans given the thank you voicemail messages they had received. They described unintended positive outcomes of the cards, including having the spouse of a veteran receiving cards call to let them know the veteran had died (not by suicide) and that this allowed them the opportunity to ensure that the spouse was taken care of and had all they needed from the VA side.

This pilot implementation program evaluation was not without limitations. A key limitation is that this was a single-site pilot which limits generalizability to other settings. However, the pilot produced a process to make decisions that may be different at other settings. Limitations related to the quantitative data on CC's effectiveness included the 6-month timeframe for analysis of effectiveness outcomes and potential limitations to the quantitative analysis described above. Regarding qualitative analysis, there was a lack of qualitative interviews with staff following implementation due to the COVID-19 pandemic. The veteran qualitative interview and survey were combined, so the number of veterans surveyed was limited. As this was program evaluation, we did not collect self-report measures from veterans. Despite these limitations, the project had numerous strengths. Strengths included leadership support at the local, regional, and national levels; stakeholder participation in planning across the system, including veterans and experts in CC and facilitation; use of an existing implementation planning guide template; use of an implementation strategy that could be customized to setting and stakeholder needs; and an evidence-based practice that is strongly supported by both research and policy.

This project demonstrated that it is feasible to implement CC in a VA ED setting in a way that allows for reach and minimizes provider burden. The project resulted in a variety of tools that can support implementation in other settings. This planning and pilot implementation informed a subsequent, currently ongoing grant-funded implementation project to spread CC to 28 VA EDs. This larger implementation project will allow for longer observation periods to evaluate the impact of being mailed the full CC intervention and to evaluate implementation in a variety of different VA EDs. This project and manuscript contribute to the field of implementation science by providing description of a process to adapt an intervention and plan implementation in a way that values both the research base and local stakeholder input and values. This process could be used with other interventions and settings using the Behavioral Health QUERI implementation planning guide template (30).

## Data Availability Statement

The original contributions presented in the study are included in the article/Supplementary Files, further inquiries can be directed to the corresponding author/s.

## Ethics Statement

The studies involving human participants were reviewed and approved by Central Arkansas Veterans Healthcare System (CAVHS) Institutional Review Board (IRB). Written informed consent for participation was not required for this study in accordance with the national legislation and the institutional requirements.

## Author Contributions

SL, JK, KC, and JA designed the study. SL, SJ, JK, NC, BM, MR, and KC participated in the planning phase. SL and SJ provided facilitation to the facility. TA, SM, JW, and NC collected, coded, and analyzed the qualitative data. JAP, MB, and JP analyzed and interpreted quantitative data. DJ and RS updated the literature search and contributed to writing the background. SL, SJ, and JAP were major contributors to the writing of the manuscript. All authors contributed to the article and approved the submitted version.

## Funding

This project was supported by funding from Central Arkansas Veterans Healthcare System, the Implementing Caring Contacts for Suicide Prevention in Non-Mental Health Settings Quality Enhancement Research Initiative (QUERI) grant (PII 18-195) awarded to the first author, and the Team Based Behavioral Health QUERI grant (QUE 15-289) awarded to the third author through the Department of Veteran Affairs.

## Author Disclaimer

This work was authored as part of the Contributor's official duties as an Employee of the United States Government and is therefore a work of the United States Government. In accordance with 17 U.S.C. 105, no copyright protection is available for such works under U.S. Law. Portions of these findings were presented in an educational cyberseminar in the VA's HSR&D Cyberseminar Lecture Series in March 2020 and as a research conference presentation at the American Association of Suicidology in April 2020. The views expressed in this paper are those of the authors and do not necessarily reflect the position or policy of the United States Department of Veterans Affairs (VA), Veterans Health Administration (VHA), or the United States Government.

## Conflict of Interest

The authors declare that the research was conducted in the absence of any commercial or financial relationships that could be construed as a potential conflict of interest.

## Publisher's Note

All claims expressed in this article are solely those of the authors and do not necessarily represent those of their affiliated organizations, or those of the publisher, the editors and the reviewers. Any product that may be evaluated in this article, or claim that may be made by its manufacturer, is not guaranteed or endorsed by the publisher.

## Footnotes

^1^Name has been changed.
