# Supplementary material for: Adapting Caring Contacts for Veterans in a Department of Veterans Affairs Emergency Department: Results From a Type 2 Hybrid Effectiveness-Implementation Pilot Study
Source: Front Psychiatry. 2021 Oct 13;12:746805. doi: 10.3389/fpsyt.2021.746805 (PMC8548725; doi:10.3389/fpsyt.2021.746805)
Supplement: Supplementary file 1 [file Data_Sheet_1.docx]

**Appendix 1**

# Caring Contacts from the Emergency Department/Urgent Care Center

Implementation Planning Guide

**Site:**

**Identified Lead:**

| **Roles/Tasks** | **Actionable Items/Examples*** | **Current Status/Potential Barriers/Notes** | Plan (including timeframe) | **Who’s in Charge?** | **Metrics and How you define success for each item** |
| --- | --- | --- | --- | --- | --- |
| **A. Identify Participating Staff** | - Identify CC Champion - Identify CC Specialist - Determine staff to participate in planning: e.g., physicians, nurses, social workers, health techs, admin staff, SPCs, etc. |  | *We strongly encourage participation of as many providers and staff as possible in planning.* |  |  |
| **B. Determine Content of Caring Contacts** | - Use existing CC ED templates - Author or signature of CC - Nurse - Social worker - Physician - Care team - Combination - Local contact information/ phone numbers (required) - Dedicated CC ED phone line & mailbox - Logos - Local VA (required) - Veterans Crisis Line (not required) - Other |  | *We strongly recommend using existing CC ED templates for cards that were developed with VA provider, Veteran, and expert input to be appropriate for outreach following an ED visit.*  *Having one LIP and the care team (e.g., Emergency Department or Emergency Team) is suggested, as in templates.*  *Do not include messages that are a demand (e.g., please call us, attend your appointments). You should put a number to call if someone would like to reach out (e.g., we are available if you need assistance).*  *Follow* [*VA style guide*](https://www.va.gov/playbook/downloads/VHA_Style_Guide_508.pdf) *around font (i.e., Georgia, Calibri, Myriad Pro) and size (not smaller than 11 pt). See style guide for other info on use of logos, clipart, etc.*  *Handwritten messages are not needed, per research.* |  |  |
| **C. Determine Start Date** | - Identify start date for the implementation of this plan. - It is ok change the start date after it was selected due to kinks in the plan. |  | *We recommend doing this ASAP after beginning to develop your plan.* |  |  |
| **D. Engage & Train Staff** | - Determine which staff will be involved in Implementation Planning process (a meeting to complete the rest of this sheet). - Educate all staff through meetings and communications. - In-services with nursing staff - See section K for aligning with other CC programs. - Continue to engage and educate leadership. - Ensure all staff completes all brief CC training as needed. Training includes rationale for CC, how Veterans are identified, and local SOPs. |  | *We recommend that staff is trained prior to your start date.*  *Include ED, mental health, and primary care staff. See Training Plan Template.* |  |  |
| **E. Determine Who to Receive CC** | - Identify Veteran population to receive CC - Any other CC program? See item K. - All who screen positive in the ED/UCC through the CSSRS, CRSE, or ICD code |  | *We recommend including all Veterans who screen positive in the ED/UCC through the CSSRS, CRSE, or ICD code; this data can be pulled easily on the SPED dashboard.*  *VA* [*clinical practice guidelines*](https://www.healthquality.va.gov/guidelines/MH/srb/VADoDSuicideRiskFullCPGFinal5088212019.pdf) *recommend CC “after psychiatric hospitalization for suicidal ideation or a suicide attempt.”* |  |  |
| **F. Determine Schedule of CC** | - Use timing of CC - Send over the course of a year - Schedule based on research studies: - Week 1; Months 1-4, 6, 8, 10, 12; Birthday - Veterans Day (optional) |  | *We strongly recommend using this schedule, as it is based on Veteran feedback and research studies.* |  |  |
| **G. Determine Who Administers** | - Method to administer CC - Postcard in envelope - Determine who administers - MSA or PSA - Health tech - Social worker - Other |  | *The person who administers CC (sending and documenting) will need access to the electronic medical record. Caring Contact Specialist.* |  |  |
| **H. Determine Logistics of Sending CC** | - Obtaining correct Veteran contact information - Utilize return address on card - Identify local standards for mailing (e.g., envelopes with VA logo only?) - Determine capacity for mailing and providing postage for greeting card envelopes - Identify resources and processes for printing |  | *Consider using your print shop to print cards and hand write Veteran names when sending.* |  |  |
| **I. Determine Who Will Document in EMR** | - Method of documentation - Local note template - Identify who will document - Person printing/mailing CC - Provider - Other clinical staff - Administrative support - Other - When will documentation happen - When CC is mailed |  | *We strongly recommend that the person printing/mailing the CC document when the card is prepared and mailed. This will allow them to see any relevant changes in CPRS.*  *Consistent with VA policy, documentation of each CC contact in medical record is required.* |  |  |
| **J. Reporting Caring Contacts Use** | - Identify reports to document number of Veterans served by CC |  | *Consider using the excel spreadsheet from facilitators.* |  |  |
| **K. Align with Other Caring Contacts Programs** | - Determine what other CC programs exist - High risk list - REACH VET - Other |  | *Determine if and how your facility will address multiple CC programs (e.g., if Veteran is receiving REACH VET CC, they would not receive CC ED).*  *Note: CC are being sent from the Veterans Crisis Line. Consider these independent from your facility.* |  |  |
